# Efficacy and Safety of Pneumatic Lithotripsy for Ureteric Stones: A Retrospective Analysis From Sudan

**DOI:** 10.7759/cureus.98723

**Published:** 2025-12-08

**Authors:** Mohammed Elsiddig, Mohammed Hassan

**Affiliations:** 1 Urology, Military Hospital, Khartoum, SDN

**Keywords:** endourology, pneumatic lithotripsy, stone treatment, ureteric stone, ureteroscopy

## Abstract

Introduction

Ureteric stone management has undergone a transformation since the advent of different types of lithotripters. Although holmium: yttrium-aluminum-garnet laser lithotripsy has been considered the gold standard of choice for several centers, it is limited in availability in underdeveloped/low-resource areas. Pneumatic lithotripsy (PL) is still a relatively inexpensive and widely available modality. The purpose of this study is to evaluate the effectiveness and safety of PL as an alternative to laser lithotripsy in low-income settings.

Methods

Retrospective data from 215 patients treated at our center using ureteroscopic PL for ureteric stones between January 2020 and January 2023 were used to conduct this analysis. Patient demographic information, characteristics of the stones (location and size), operating time, stone-free rate (SFR) of the patients, complications, and length of hospitalization were all documented and assessed.

Results

The average age of the patients included in the study was 42.5 ± 11.8 years old; 147 (68.4%) patients were men. Most of the stones were in the lower ureter (153, 71.2%); the mean diameter of the stones was 12.1 ± 3.5 mm. The overall initial SFR was 192 (89.3%). Patients had a higher SFR for stones in the lower ureter (145, 94.7%) than the middle (31, 85.2%) and upper ureter (20, 76.9%) (p<0.05). The mean operative time was 45.2 ± 15.7 minutes. Complications were experienced by 45 (20.9%) patients. Minor complications were observed in 40 (18.6%) patients, most commonly postoperative hematuria and colic. Major complications were rare (5, 2.3%) and included two cases of ureteral perforation and three cases of steinstrasse, which required intervention.

Conclusion

PL provides effective and safe treatment of ureteric stones, especially stones in the lower ureter. Due to cost, equipment availability, and other factors related to care in resource-poor countries such as Sudan, PL continues to be a valuable and appropriate first-line treatment for ureteric stones. Therefore, PL has secured its place in the contemporary urologic treatment arsenal as a reliable and affordable alternative to laser lithotripsy.

## Introduction

Urolithiasis, which has an increasing global incidence and prevalence, is a major urological problem [1]. Of those patients who present to emergency departments with urolithiasis, many suffer from acute renal colic; therefore, they require immediate intervention to avoid obstructive complications (such as infection and/or loss of renal function) [2]. Advances in endourology have transformed the way we manage patients with ureteric stones, and with spontaneous passage being less likely for larger stones and/or those that cannot pass after non-invasive therapy, the initial treatment of choice now involves a combination of semi-rigid ureteroscopy (URS) and intracorporeal lithotripsy for stone fragmentation [2,3].

The selection of the intracorporeal lithotripter will impact both the success of URS and the overall cost of the procedure. Due to the versatile nature of the laser, including its capability to break down a wide variety of stone types without significant retropulsion, the holmium: yttrium-aluminum-garnet (Ho: YAG) laser is considered the "gold standard" for intracorporeal lithotripsy [4,5]. However, the cost associated with purchasing the equipment, ongoing service/maintenance fees, and replacement costs for the laser fibers limit the ability of this technique to be universally applied throughout the world, especially in low-income countries such as Sudan [6,7].

A second type of mechanical lithotripter is pneumatic lithotripsy (PL). PL generates a ballistic force using compressed air to fragment stones and has several advantages over laser lithotripsy, including long-term durability, lower operational costs, and greater reliability [8]. Although PL has many benefits, there are also concerns that the potential for stone retropulsion into the kidney (especially for stones located in the upper part of the ureter) and possible complications such as an increased risk of ureteral mucosal injury could result in a longer hospital stay for the patient [9-11]. As a result of these limitations, PL has largely fallen out of favor in high-income countries, and in those countries, it is often replaced with laser lithotripsy.

Due to the limited availability of Ho: YAG laser technology in Sudan, as well as the limited access to that technology for much of the population, PL continues to be the most commonly used intracorporeal lithotripter for patients undergoing URS at the majority of public and teaching hospitals in Sudan. There is little published information about the results of comparing PL with laser lithotripsy in Sudan, where patient demographics, stone characteristics, and the number of surgical procedures may be different than those in developed countries.

Therefore, the primary purpose of this study is to perform a retrospective review of evaluating the effectiveness, safety, and clinical outcome of ureteroscopic PL for the treatment of ureteric stones as an alternative to laser lithotripsy in a low-income setting. Secondary goals of the study were to assess the complication rates and operative time for this technique in order to provide further insight into whether this technology still has a role in the current urologic practice.

## Materials and methods

Study design and setting

A single-center retrospective observational study was conducted at the Urology Department of Omdurman Military Hospital, which is one of the main tertiary care centers in Sudan.

Study cohort

Adult patients (>18 years old) who had undergone semi-rigid URS with PL for a single, radiopaque ureteric stone during the period from January 1, 2020 to January 31, 2023 were included in this study. Patients excluded from the cohort were those with other renal stones, radiolucent stones, or known ureteral strictures.

Variables and data collection

Demographic data, including age and gender, along with the characteristics of the stones (laterality - left or right; location - upper ureter: above the sacroiliac joint, middle ureter: the area surrounding the sacroiliac joint, or lower ureter: below the sacroiliac joint; size - the maximum size of the stone as determined through non-contrast computed tomography were extracted from the medical records and operative theater logs. Operative data, including the total operative time and the placement of a double-J stent, either pre- or post-operatively, were also extracted.

Outcome measures

The primary outcome was the stone-free rate (SFR) for the patients. Patients were classified as having been successfully treated if their follow-up plain abdominal radiograph, performed at 4 weeks post-procedure, did not show any residual stone fragments larger than 3 mm. Additional secondary outcomes included an assessment of the incidence of intraoperative complications, including mucosal injuries or ureteral perforations, and postoperative complications that occurred within 30 days. In addition, the length of the postoperative hospitalization was also recorded.

Surgical procedures

All of the surgical procedures were performed under spinal or general anesthesia by experienced urologists or senior registrars working directly under supervision. A consistent methodology was used for each case. Each case involved a cystoscopic examination followed by the placement of a guidewire into the renal pelvis. Following this, a semi-rigid ureteroscope was inserted into the ureter under direct visualization. The stone was identified and fragmented using a 2.0 mm pneumatic lithotripter probe under constant saline irrigation. The resultant stone fragments were then retrieved from the ureter using a grasper or a basket extractor. The decision to insert a double-J stent was made intraoperatively by the surgeon based upon factors such as the degree of ureteral edema, the extent of mucosal trauma, and the overall operative time. The stent was removed cystoscopically approximately 2 weeks after the original surgery.

Statistical analysis

IBM SPSS Statistics for Windows version 26.0 (IBM Corp., Armonk, NY) was used to perform the statistical analysis of the data. Means and standard deviations were reported for the continuous variables, while categorical variables were reported as frequency counts and percentage values. The chi-square test was used to assess differences among categorical outcomes, for example, the SFR among different stone localizations. Statistical significance was set at p-value <0.05.

## Results

We analyzed 215 eligible patients to evaluate treatment outcomes. The mean age of the study population was 42.5 years, and the majority were males (147, 68.4%). Stones were more frequently located on the left side (118, 54.9%). The lower ureter was the most common site (153, 71.2%), followed by the middle (36, 16.7%) and upper ureter (26, 12.1%). The average stone size was 12.1 mm. The demographics and clinical details of our study subjects at baseline are outlined in Table 1.

**Table 1:** Patient and stone characteristics at baseline SD: Standard deviation.

Characteristic	Value
Mean age (years ± SD)	42.5 ± 11.8
Gender	n (%)
Male	147 (68.4%)
Female	68 (31.6%)
Stone laterality	n (%)
Left	118 (54.9%)
Right	97 (45.1%)
Stone location	n (%)
Upper ureter	26 (12.1%)
Middle ureter	36 (16.7%)
Lower ureter	153 (71.2%)
Mean stone size (mm ± SD)	12.1 ± 3.5

Table 2 depicts SFR by stone location. We achieved an overall SFR of 192 (89.3%) immediately after one treatment session. We also found a significant relationship between the site of the stone and the SFR (p<0.05). The SFRs were as follows: lower ureter: 145 (94.7%), middle ureter: 31 (85.2%), and upper ureter: 20 (76.9%).

**Table 2:** Stone-free rate by stone location Statistical test: Chi-square test, χ²=15.82, p<0.05

Stone location	Total patients	Stone-free patients	Stone-free rate (%)
Upper ureter	26	20	76.90%
Middle ureter	36	31	85.20%
Lower ureter	153	145	94.70%
Overall	215	192	89.30%

Table 3 lists the intraoperative and postoperative complications that occurred during this study. All 45 (20.9%) patients experience complications. Minor complications were experienced by 40 (18.6%) patients. Hematuria lasting less than a week was the most common complication at 20 (9.3%), and post-operative colic treated with oral analgesics was second at 15 (7.0%). Five patients experienced major complications, two patients had intraoperative ureteral perforations treated with prolonged stenting, and three had steinstrasse that required repeat URS for removal of fragments. No serious complications or deaths resulted from the procedures. We also found that the incidence of clinically significant stone retropulsion into the kidney was very low at 6 (2.8%), and all were successfully treated with subsequent shock wave lithotripsy. Average operative time was 45.2 ± 15.7 minutes. We inserted a double-J stent in 189 (87.9%) patients postoperatively. The average length of hospitalization postoperatively was 1.4 ± 0.7 days.

**Table 3:** Intraoperative and postoperative complications URS: Ureteroscopy; UTI: Urinary tract infection.

Complication	n (%)	Management
Intraoperative		
Mucosal injury	12 (5.6%)	Conservative stenting
Ureteral perforation	2 (0.9%)	Prolonged stenting
Stone retropulsion	6 (2.8%)	Shock wave lithotripsy
Postoperative (within 30 days)		
Transient hematuria	20 (9.3%)	Observation
Postoperative colic	15 (7.0%)	Oral analgesics
Febrile UTI	8 (3.7%)	Antibiotics
Steinstrasse	3 (1.4%)	Repeat URS

## Discussion

This retrospective study from a single tertiary center in Sudan shows that pneumatically assisted lithotripsy is a very successful method to treat ureteric stones. An overall success rate of 89.3% was achieved in this study. Success rates of PL reported in previous literature ranged between 85% and 95%; therefore, results of this study support literature findings [12-14].

The primary advantage of PL lies in its superior cost-effectiveness and low equipment maintenance costs compared to laser-based modalities. The initial purchase price of the PL machine is much less than the price of the Ho: YAG laser machine. Additionally, costs associated with reusable probes of PL machines are relatively low [13]. For low- and middle-income countries, both factors are extremely important. Since budgets allocated for the medical equipment are very limited, the economic viability of PL is one of its primary benefits.

Consistent with prior studies, our results revealed key disadvantages of PL, particularly its lower efficacy in treating upper ureteric stones (20, 76.9%) compared to lower ureteric stones, which represents one of the primary drawbacks of PL. Major complications of PL, which were observed in this study, occurred at a rate of 2.3%, which is similar to the rates reported in other studies, which typically vary between 1% and 5% [12,15]. 

PL appears to retain an important role in the treatment of ureteric stones in Sudan and other resource-constrained healthcare environments. High success rates of PL, especially for lower ureteric stones, combined with the cost-effectiveness of the technology, ensure that this modality will continue to play an essential role in the management of ureteric stones in resource-poor countries. Therefore, future prospective and randomized studies comparing PL with laser lithotripsy in our population will provide additional valuable information.

As a result of its retrospective nature, this study has several limitations. The retrospective design of the study may lead to potential selection and information bias. Additionally, the lack of a direct comparative arm (e.g., laser lithotripsy) limits the ability of this study to directly compare the two modalities and determine which one is superior. Finally, the lack of routine availability of stone composition data for all patients in this study may influence fragmentation efficiency.

## Conclusions

PL remains a highly effective, safe, and economically sustainable treatment for ureteric stones, particularly in the middle and lower ureter, and continues to serve as a practical alternative to laser lithotripsy in resource-limited environments. While the Ho: YAG laser represents the gold standard of care globally, the practical and cost-effective characteristics of PL secure its position as an alternative to laser lithotripsy in resource-limited countries.
